# Financial burden of older inpatients with multimorbidity in China: a cross-sectional study

**DOI:** 10.3389/fpubh.2026.1767499

**Published:** 2026-04-30

**Authors:** Bensong Xian, Seung Chun Paek, Weiyun Jin, Thammarat Marohabutr

**Affiliations:** 1 Faculty of Social Sciences and Humanities, Mahidol University, Nakhon Pathom, Thailand; 2 School of Health Management, Inner Mongolia Medical University, Hohhot, China; 3 College of Humanities Education, Inner Mongolia Medical University, Hohhot, China

**Keywords:** cross-sectional study, financial burden, multimorbidity, older inpatients, out-of-pocket expense

## Abstract

**Background:**

In China, which has the world’s largest and one of the fastest-aging populations, multimorbidity has become a major public health challenge. Older individuals with multiple chronic conditions experience poor health outcomes, increased healthcare utilization, and substantial financial pressure. This study investigates of the financial burden on older inpatients with multimorbidity.

**Methods:**

Utilizing 2022 inpatient data from a Chinese city (covering 4 secondary and 2 tertiary hospitals), this study employed descriptive statistics (means, standard deviations, medians, and interquartile ranges), comparative tests (chi-square, *t*-test, ANOVA), and multiple linear regression to identify key drivers of medical costs and out-of-pocket expenses for older inpatients with multimorbidity.

**Results:**

The study revealed a high multimorbidity rate of 73.8% among 106,202 inpatients aged ≥50 years from six Chinese hospitals in 2022. The average total hospitalization cost was 10,318 yuan, with an out of pocket expense (OOPE) of 4,558 yuan. Descriptive analysis showed the largest patient proportion was aged 60–69 years (38.4%) and most had 2–4 chronic conditions. Analysis of medical expenses identified significant variations. Patients with urban employee insurance had higher total costs but lower OOPE (24.99% proportion) compared to those with urban–rural resident insurance (50.89% proportion). Treatment in tertiary hospitals and longer hospital stays were strongly associated with higher costs. Multiple regression confirmed hospital level, length of stay, and surgical intervention were the most significant positive predictors of total costs. For OOPE, health insurance type, hospital level, and length of stay were major influencing factors.

**Conclusion:**

Multimorbidity is highly prevalent and imposes a substantial financial burden on older inpatients. Strengthening prevention, optimizing insurance design, and improving integrated care are essential for promoting healthcare equity and sustainability in China.

## 1 Introduction

The challenge of population aging is becoming increasingly significant globally. As per the 2022 World Population Prospects report from the United Nations, almost every country in the world has an increasing number and proportion of older population (1). The share of the global population aged 65 years and above is set to climb from 10% in 2022 to 16% by 2050 (1). China has the world’s largest older population and is one of the fastest-aging countries globally (2). By the end of 2022, the population aged 60 years and above had reached 280.04 million, representing 19.8% of the total populace (3). According to the China Statistical Yearbook, the population aged 50 and above accounts for 37.84% of the total population, further highlighting the magnitude of demographic aging in the country (4). Projections indicate that by 2050, the older population will make up half of China’s total population (5), potentially leading to a surge in multimorbidity cases. Chronic diseases are one of the greatest health and development challenges of the 21st century. According to World Health Organization report, chronic disease causes 41 million deaths annually, which accounts for 74% of all global fatalities (6). Every year, 17 million individuals die from chronic illnesses before reaching the age of 70, with 86% of these premature deaths happening in low- and middle-income nations. Chronic diseases disproportionately affect residents of low- and middle-income countries, where they make up over three-quarters of the global chronic disease-related deaths, totaling 31.4 million people. In China, around 88% of total deaths are linked to chronic conditions like cardiovascular diseases, cancer, chronic respiratory diseases, and diabetes. The disease burden caused by these chronic diseases constituted over 70% of the overall disease burden. Furthermore, as the population ages, the prevalence of chronic diseases among older individuals tends to be 2–3 times higher than that of the general population (7). This trend poses a significant challenge to healthcare systems and necessitates a comprehensive approach to address the growing burden of older population with chronic diseases in China.

The aging of populations and the increasing prevalence of chronic diseases are resulting in a higher number of individuals living with multiple health conditions. As lifestyles evolve, personal risk factors increase, and populations age, multimorbidity has taken the place of infectious diseases as the primary health concern among older populations. This presents a significant challenge to public health systems in all countries (8–10). Multimorbidity, defined as the coexistence of two or more chronic conditions in the same individual at the same time (11), particularly in countries with a rapidly aging population (12). In high-income countries, there is a high prevalence of multimorbidity among older individuals, typically those aged 65 and above (13), affecting at least 50 million people in the European Union (14). Multimorbidity is also becoming more prevalent in low- and middle-income nations, where already fragile healthcare systems are strained by the combined challenges of chronic and infectious diseases (12, 15). With the world transitioning into an aging society, there is a growing concern about public health issues related to increased geriatric illnesses and the subsequent rise in healthcare costs for older populations (16). The health outlook for China’s older population is far from optimistic. With advancing age, issues related to cognition, physical activity, sensory functions, as well as nutritional and psychological well-being become increasingly prominent. In China, more than 78% of older individuals grapple with multimorbidity (2). From high-income countries to low- and middle-income nations, the burden of multimorbidity is evident, straining already fragile healthcare infrastructures. As societies continue to age and chronic diseases become more prevalent, effective management of multimorbidity is crucial for promoting healthy aging and improving overall population health outcomes.

Multimorbidity in older individuals has significant implications for health outcomes and healthcare systems. Multimorbidity poses a significant challenge to healthcare systems due to its strong correlation with adverse health outcomes, increased complexity in clinical management, heightened utilization of healthcare services, and the resulting financial burden (17–21). Multimorbidity is strongly associated with adverse health outcomes, increased healthcare utilization, and substantial financial burden on both individuals and healthcare systems (22–27). In addition, the management of multimorbidity exacerbates the economic ramifications of chronic diseases for patients and their families, often leading to significant financial strain that can prevent access to essential healthcare services. This situation further compounds the health and financial challenges experienced by patients (28). In summary, multimorbidity in older individuals has affecting not only individual health outcomes but also placing a substantial burden on healthcare systems and creating economic challenges for patients and their families.

This study investigates the financial burden on older inpatients with multimorbidity, particularly focusing on the impact of out-of-pocket expenses in the context of China’s evolving healthcare system. The research findings may inform the development of health policies designed to support older individuals with multimorbidity and help reduce their financial burden.

## 2 Methods

### 2.1 Data sources

This study was conducted in a prefecture-level city in Inner Mongolia Autonomous Region, China. The city consists of several counties and banners, with a substantial older population (individuals aged 60 years and above accounting for 21.05% of the total population) and a relatively well-developed healthcare infrastructure, making it suitable for investigating the economic burden of multimorbidity among older adults.

Data were sourced from inpatient expense records of six general hospitals in this city for the year 2022. Hospital selection followed a two-stage random sampling procedure. First, to minimize selection bias, each county in the city was assigned a unique identifier, and a random number generator was used to select six counties. Second, in each selected county, if more than one general hospital was present, one hospital was randomly selected. This process yielded hospitalization data from six general hospitals across six counties, comprising 4 secondary and 2 tertiary general hospitals.

The focus on secondary and tertiary hospitals is justified as they are the primary centers for treating older inpatients with complex multimorbidity in China, as primary care institutions typically lack the capacity for such cases. Inner Mongolia is a typical representative of China border ethnic regions, characterized by vast territory, a relatively sparse population, lagging economic development, limited health resource allocation, and weaker health service capacity compared to developed coastal areas. Although this sample is not probabilistically representative of all hospitals in China, it provides an important case reference for understanding the financial burden of older inpatients with multimorbidity in China border ethnic regions—areas that remain underrepresented in existing research. Furthermore, confining the study to a single administrative area enhances internal consistency by controlling for regional variations in healthcare policies and pricing.

#### 2.1.1 Hospital system context

General hospitals in China operate under a tiered management system, with varying service capacities and functional orientations. Primary hospitals primarily serve communities, delivering basic medical and public health services. Secondary hospitals function as regional medical centers, serving multiple communities and providing technical guidance to lower-level institutions. Tertiary hospitals possess cross-regional diagnosis and treatment capabilities, serving as comprehensive centers integrating medical care, teaching, and scientific research for the diagnosis and treatment of complex and severe illnesses. The medical institutions involved in this study represent core providers within this system.

#### 2.1.2 Study population and rationale

This study focused on inpatients aged 50 years and older whose primary diagnosis was a chronic condition, aiming to analyze the economic burden of disease among patients with multimorbidity. The inclusion of the 50–59 age group is supported by both academic rationale and practical significance. Although the international community commonly defines old age as starting at 60 or 65 years, the accumulation of chronic disease risks often begins in midlife. The WHO notes that aging is characterized by the gradual accumulation of molecular and cellular damage, necessitating proactive health risk monitoring that includes middle-aged populations (29). To systematically capture the early trajectory of health transitions, authoritative studies such as the Study on Global AGEing and Adult Health (SAGE) have consistently adopted 50 years as the inclusion criterion (30). Furthermore, the 50–59 age group represents a critical window for chronic disease prevention and control that bridges middle and older age. Analyzing its economic burden can provide crucial evidence for formulating forward-looking health intervention strategies and mitigating health risks as this population transitions into advanced age.

#### 2.1.3 Case selection and definitions

Case identification was performed using ICD-10 codes based on a list of 46 chronic diseases established by Koller et al. (31). The full list of 46 chronic diseases and corresponding ICD-10 codes is provided in Supplementary Table S1. The study only included cases where a chronic disease was designated as the “primary diagnosis.” This diagnosis represents the main health problem accounting for the core treatment received and the majority of healthcare resources consumed during the specific hospitalization episode. To quantify multimorbidity, this study considered chronic conditions listed within the first five diagnoses in the medical records. Diagnoses listed sixth or beyond were not considered. The decision to retain only the first five diagnoses is based on clinical practice, as these typically represent the most clinically significant conditions driving the current admission and account for the majority of healthcare resource consumption. Diagnoses listed sixth or beyond are often less relevant to the primary reason for hospitalization and were therefore excluded.

#### 2.1.4 Unit of analysis

The unit of analysis in this study is the hospitalization episode. Each record represents a single inpatient admission. In cases where the same patient had multiple admissions during the study period, each admission was treated as an independent observation. This approach may violate the independence assumption in regression models; however, given that repeat admissions accounted for a small proportion of the total sample and the focus of this study is on episode-level costs and resource utilization, we did not apply clustering adjustments. Future studies with longitudinal patient-level data could further explore this issue.

### 2.2 Variable selection

#### 2.2.1 Dependent variable

The study used two dependent variables, namely medical expenses and out-of-pocket expenses (OOPE) of inpatients. The two variables are complementary. Medical expenses reveal the magnitude of costs generated by multimorbidity, while OOPE indicates the extent to which these costs are transferred to patients. When analyzed together, they allow a comprehensive understanding of whether high patient burdens are primarily driven by high overall medical spending, insufficient insurance coverage, or both. These two variables capture both system-level resource consumption and patient-level financial burden, offering a balanced basis for policy recommendations. Table 1 shows the variables applied in this study.

**Table 1:** Variables applied in this study.

Variable	Item	Description	Type
Dependent variable	Medical expenses	≥0 yuan	Continuous
	Out-of-pocket expenses	≥0 yuan	Continuous
Independent variable	Age	50–59 years 60–69 years 70–79 years 80 years and above	Categorical (Ordinal data)
	Number of chronic diseases	2 chronic diseases 3 chronic diseases 4 chronic diseases 5 or more chronic diseases	Categorical (Ordinal data)
	Health insurance scheme	Basic Medical Insurance for Urban and Rural Residents Basic Medical Insurance for Urban Employees	Categorical (Nominal data)
Control variable	Hospital level	Secondary general hospitals Tertiary general hospitals	Categorical (Ordinal data)
	Length of hospital stay	1–5 days 6–10 days More than 10 days	Categorical (Ordinal data)
	Surgical intervention	Had surgery Did not have surgery	Categorical (Nominal data)

##### 2.2.1.1 Medical expenses

Medical expenses refer to all expenses incurred by patients during inpatient medical treatment, including medical service fees, examination fees, medication expenses, laboratory fees, surgical costs, nursing fees, and bed fees. This variable reflects the total cost of hospitalization and captures the overall resource consumption of the healthcare system and the economic burden from a societal perspective.

All monetary values in this study represent actual hospital charges (i.e., the total costs billed by hospitals, not the amounts paid out-of-pocket by patients), and are reported in nominal 2022 Chinese yuan (CNY), as no inflation adjustment was applied.

##### 2.2.1.2 Out-of-pocket expenses

Out-of-pocket expenses for inpatients are the costs that inpatients pay directly to the hospital during their hospital stay, which are not covered by health insurance payments. These expenses reflect the actual amount paid by the patient. The advantage of this variable is that it directly indicates the financial hardship faced by inpatients and their families, making it essential for assessing affordability and equity.

#### 2.2.2 Independent variables

##### 2.2.2.1 Age

Age is categorized into four groups: “50–59 years,” “60–69 years,” “70–79 years,” and “80 years and above.” It is measured as an ordinal categorical variable. Grouping age into ten-year intervals enhances statistical stability and comparability, and aligns with common demographic reporting practices. It also aligns with common age grouping methods in real life, aiding in capturing different age group characteristics and trends.

##### 2.2.2.2 Number of chronic diseases

This study analyzed chronic disease diagnoses from the first to the five for determining comorbidity quantity, with diagnoses from the fifth onward not being considered. The first five diagnoses typically represent the most clinically significant conditions and account for the majority of healthcare resource consumption during hospitalization. Investigation of the first five diagnoses can enhance a better understanding of their impact on the economic burden of hospitalized patients. The number of chronic diseases is categorized into four groups: “2 chronic disease,” “3 chronic diseases,” “4 chronic diseases” and “5 or more chronic diseases.” The number of chronic diseases is measured as an ordinal categorical variable.

##### 2.2.2.3 Health insurance scheme

China’s healthcare security system mainly consists of Basic Medical Insurance for Urban and Rural Residents and Basic Medical Insurance for Urban Employees. This study categorizes the health insurance scheme into “Basic Medical Insurance for Urban and Rural Residents” and “Basic Medical Insurance for Urban Employees.” The health insurance scheme is measured as a nominal categorical variable.

The Urban Employee Basic Medical Insurance covers formally employed individuals, with premiums jointly contributed by employers and employees. It typically offers a higher reimbursement rate and a broader benefits package. In contrast, the Urban and Rural Resident Basic Medical Insurance covers non-employed residents, including children, students, and the people not in formal employment, with lower premiums and correspondingly lower reimbursement rates. These structural differences in financing and benefit design contribute directly to the observed disparities in out-of-pocket expenses between the two schemes.

#### 2.2.3 Control variables

##### 2.2.3.1 Hospital level

In China, general hospitals are categorized into three levels: primary general hospitals, secondary general hospitals, and tertiary general hospitals. However, primary general hospitals, which do not admit inpatients, are not included as subjects in this study. Therefore, the hospital level is categorized into two groups: “Secondary general hospitals” and “Tertiary general hospitals.” It is measured as an ordinal categorical variable.

##### 2.2.3.2 Length of hospital stay

Length of hospital stay is categorized into three groups: “1–5 days,” “6–10 days,” and “More than 10 days.” It is measured as an ordinal categorical variable. Categorizing hospital stay into these three groups balances clinical relevance with statistical reliability.

##### 2.2.3.3 Surgical intervention

Surgical intervention is categorized into two groups: “Had surgery” and “Did not have surgery.” It is measured as a nominal categorical variable.

### 2.3 Statistical methods

Descriptive statistical method was employed to summarize the basic characteristics of the study sample. For continuous variables, means and standard deviations were calculated to describe the central tendency and dispersion, and medians with interquartile ranges (IQR) were additionally reported to better capture the distribution of cost data, which were right-skewed. The chi-square test was used for comparisons between groups of categorical variables. For continuous variables, independent sample *t*-tests or one-way analysis of variance (ANOVA) were conducted as appropriate.

Furthermore, multiple linear regression models were applied to explore the associations among variables. The models yielded an adjusted R^2^ of 0.312 for medical expenses and 0.298 for out of pocket expenses. Cases with missing values for any variable were excluded from the analysis (complete case analysis). Of the initial 106,202 hospitalized patients aged 50 years and older, 27,809 were excluded because they either did not have a chronic condition as their primary diagnosis or had only one chronic condition. The final analytic sample consisted of 78,393 patients with multimorbidity (≥2 chronic conditions). All statistical analyses were performed using SPSS version 29.0, with a two-tailed *p*-value of less than 0.05 considered statistically significant.

#### 2.3.1 Justification for the use of multiple linear regression

In addition to the descriptive and comparative analyses, multiple linear regression was employed to identify factors independently associated with medical costs and out of pocket expenses. The rationale for this choice is as follows. The study aims to assess the independent effects of multiple predictor variables while statistically controlling for potential confounders such as hospital level, length of stay, and surgical operation. Multiple linear regression is the standard methodology designed to analyze linear relationships between several predictors and a single continuous outcome variable. It allows for the direct estimation of partial regression coefficients (B values), providing a clear estimate of the impact of each factor after adjusting for the influence of all other variables in the model.

This study includes over 78,000 hospitalized patients with multimorbidity. This substantial sample size forms a critical foundation for the application of multiple linear regression. According to the Central Limit Theorem, under large-sample conditions, the sampling distribution of parameter estimates (coefficients) will approximate normality even if the original distribution of the dependent variable (medical costs) deviates from normality. This ensures the validity of hypothesis testing (e.g., *t-*tests, F test) and the reliability of statistical inference (32).

The study acknowledges that healthcare cost data often exhibit a right-skewed distribution. Nevertheless, multiple linear regression remains applicable and yields stable results in this context for the following reasons. The primary focus of this study is to identify influencing factors rather than to generate precise individual-level cost predictions. The regression coefficients reflect the average marginal effects of the independent variables. The large sample size effectively stabilizes estimates and mitigates the disproportionate influence of outliers. As a sensitivity analysis, the study calculated robust standard errors to correct for potential heteroscedasticity (33). Furthermore, the study verified that the variance inflation factors (VIFs) for key independent variables were all close to 1, indicating minimal multicollinearity and stable model estimates (34).

The unstandardized coefficients (B) obtained from multiple linear regression offer an intuitive economic interpretation (e.g., “holding other factors constant, patients with a specific insurance scheme have, on average, X Yuan less in out of pocket expenses”). Presenting the effect size in absolute terms, as opposed to relative ratios provided by some alternative models, yields evidence that is more direct and actionable for health policymakers quantifying disease-related financial burden and evaluating policy interventions (35).

In conclusion, considering the research objectives, data characteristics, and sample size, multiple linear regression represents an appropriate and robust statistical tool for this analysis.

### 2.4 Ethical considerations

The study protocol was reviewed and approved by the Medical Ethics Committee of Inner Mongolia Medical University (Approval No. YKD202402148) and the Office of the Committee for Research Ethics (Social Sciences) at Mahidol University, Thailand (Approval No. 2024/056.1806). As the study employed a retrospective design utilizing a fully anonymized and de-identified dataset, both committees granted a formal waiver for the requirement of obtaining individual patient informed consent.

## 3 Results

In 2022, among the 106,202 hospitalized patients aged 50 years and above from sampled six hospitals in China, 78,393 (73.8%) were diagnosed with multimorbidity. It is important to note that this proportion reflects the prevalence of multimorbidity within the sample of inpatients whose primary diagnosis was a chronic condition, rather than an estimate of population-level multimorbidity prevalence. The average total hospitalization cost for patients with multimorbidity was 10,318 yuan, with an average out-of-pocket expense of 4,558 yuan. The following sections present a detailed analysis of the characteristics, economic burden, and influencing factors associated with multimorbidity in these older inpatients.

### 3.1 Descriptive statistics of inpatients by demographic characteristics

**Figure 1:**
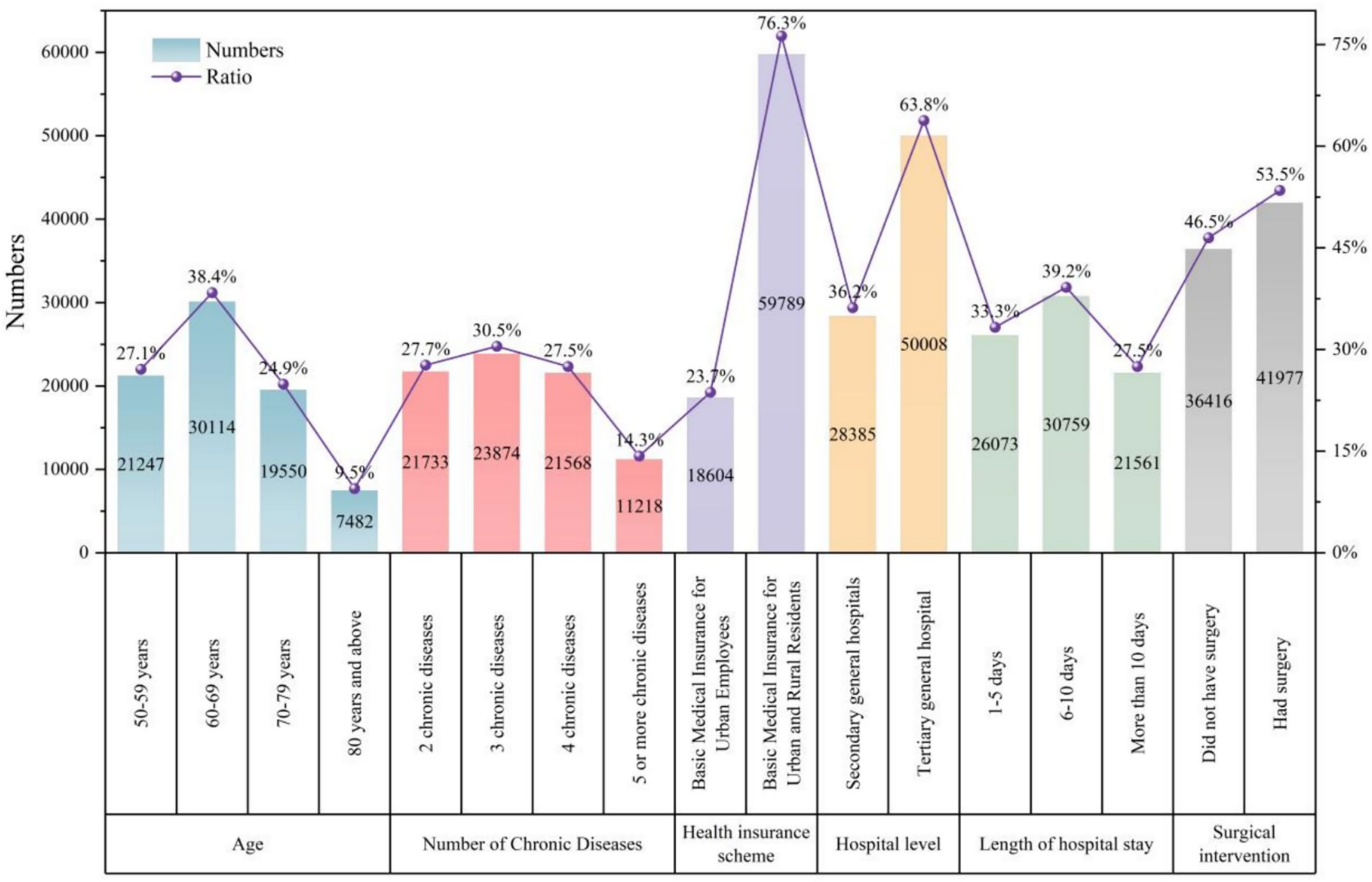
Descriptive statistics of inpatients by demographic characteristics.

In the six sampled hospitals, the characteristics of inpatients aged 50 years and above with multimorbidity were as follows. In terms of age distribution, inpatients aged 60–69 years accounted for the largest proportion (38.4%), followed by those aged 50–59 years (27.1%) and 70–79 years (24.9%), while inpatients aged 80 years and above constituted the smallest group (9.5%). Regarding the number of chronic diseases, inpatients with three chronic diseases accounted for the highest proportion (30.5%), followed by those with two (27.7%) and four (27.5%) chronic diseases, while those with five or more chronic diseases accounted for a relatively smaller proportion (14.3%). Overall, inpatients with two to four chronic diseases represented about two-thirds of the study population. In terms of health insurance coverage, most inpatients were enrolled in the basic medical insurance scheme for urban and rural residents (76.3%), while only 23.7% were covered by the urban employee scheme. Concerning hospital level, 63.8% of inpatients sought care in tertiary general hospitals, while 36.2% were treated in secondary general hospitals. The length of hospital stay was most commonly 6–10 days (39.2%), followed by 1–5 days (33.3%) and more than 10 days (27.5%). In addition, 53.5% of patients underwent surgical procedures, while 46.5% did not. Figure 1 shows descriptive statistics of inpatients by demographic characteristics.

### 3.2 Analysis of medical expenses and OOPE by demographic variables

**Figure 2:**
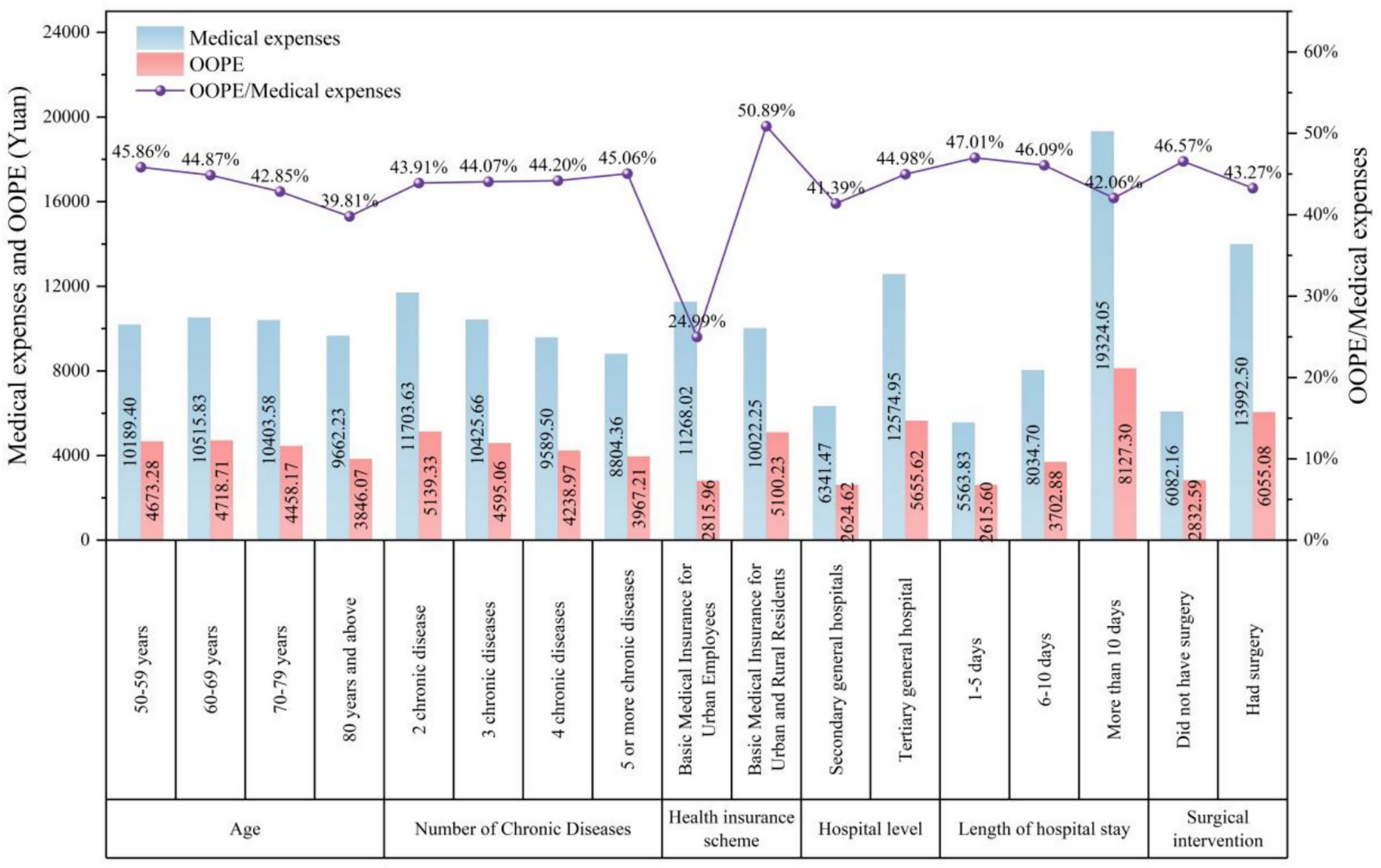
Medical expenses and OOPE per person across different variables.

Medical expenses and OOPE varied among patients with different demographic and clinical characteristics. In terms of age, the highest total costs were observed in the 60–69 years group (10,515.83 yuan), closely followed by the 70–79 years group (10,403.58 yuan). The peak OOPE was also found in the 60–69 years group (4,718.71 yuan). The proportion of OOPE showed a steady decreasing trend with age, declining from 45.86% in the youngest group (50–59 years) to 39.81% in the oldest group (aged 80 and above). Figure 2 shows medical expenses and OOPE per person across different variables.

Regarding the number of chronic diseases, inpatients with two chronic diseases had the highest total costs (11,703.63 yuan) and OOPE (5,139.33 yuan). Inpatients with two chronic diseases had the highest total costs. This pattern may be driven by treatment intensity, as patients with two conditions often receive more aggressive interventions than those with more complex multimorbidity, who may be managed more conservatively. Medical expenses and OOPE declined as the number of chronic diseases increased, although the proportion of OOPE rose slightly, reaching 45.06% among inpatients with five or more diseases.

Clear disparities were observed across health insurance schemes. Inpatients with urban employee insurance had higher medical expenses (11,268.02 yuan) but much lower OOPE (2,815.96 yuan), with a proportion of 24.99%. In contrast, inpatients with urban and rural resident insurance had lower total costs (10,022.25 yuan) but substantially higher OOPE (5,100.23 yuan), representing 50.89% of the total.

By hospital level, inpatients treated in tertiary hospitals incurred significantly higher medical expenses (12,574.95 yuan) and OOPE (5,655.62 yuan) compared with those in secondary hospitals (6,341.47 yuan and 2,624.62 yuan, respectively).

Hospitalization duration was also strongly associated with costs. Inpatients hospitalized for more than 10 days had the highest medical expenses (19,324.05 yuan) and OOPE (8,127.30 yuan), while the highest proportion of OOPE was observed among inpatients with shorter stays of 1–5 days (47.01%).

Finally, surgical inpatients had markedly higher medical expenses (13,992.50 yuan) and OOPE (6,055.08 yuan) than non-surgical inpatients (6,082.16 yuan and 2,832.59 yuan, respectively). However, the proportion of OOPE was lower among surgical inpatients (43.27%) compared with non-surgical ones (46.57%).

### 3.3 Analysis of factors influencing medical expenses across various demographics

The analysis of medical expenses among older inpatients revealed significant differences across age groups, number of chronic diseases, type of health insurance, hospital level, length of hospital stay, and surgical intervention. Table 2 shows the differences in medical expenses per person across various variables. By age group, the average medical cost was 10,189.40 yuan for inpatients aged 50–59 years, 10,515.83 yuan for those aged 60–69 years, 10,403.58 yuan for those aged 70–79 years, and 9,662.23 yuan for inpatients aged 80 years and above. The median values showed a similar pattern, with the highest median observed in the 60–69 age group (6,165.78 yuan), and the interquartile ranges (IQRs) indicated substantial variability across all age groups. Analysis of variance indicated that these differences were statistically significant (*F* = 7.55, *p* < 0.001), suggesting that medical expenses differed across age groups but do not increase monotonically with age.

**Table 2:** Differences in medical expenses per person across various variables.

Variable	Groups	Frequency	Median (Quartiles)	Mean	Standard deviation	*F/t*	*P*
Age	50–59 years	21,247	6,163.55 (7,004.90)	10,189.40	14,476.58	7.55 (*F*)	<0.001
	60–69 years	30,114	6,165.78 (7,226.47)	10,515.83	14,445.94		
	70–79 years	19,550	6,010.14 (6,918.95)	10,403.58	15,357.00		
	80 years and above	7,482	5,934.73 (6,065.91)	9,662.23	14,498.05		
Number of chronic diseases	2 chronic diseases	21,733	6,316.80(8,514.93)	11,703.63	17,875.28	122.78 (*F*)	<0.001
	3 chronic diseases	23,874	6,190.47 (7,198.73)	10,425.66	14,981.85		
	4 chronic diseases	21,568	6,043.54 (6,467.22)	9,589.50	12,540.12		
	5 or more chronic diseases	11,218	5,747.15 (5,316.07)	8,804.36	10,131.55		
Health insurance scheme	Basic medical insurance for Urban Employees	18,604	6,625.82 (7,595.53)	11,268.02	17,088.77	9.06 (*t*)	<0.001
	Basic medical insurance for urban and rural residents	59,789	5,930.16 (6,797.63)	10,022.25	13,850.71		
Hospital level	Secondary general hospitals	28,385	4,416.07 (3,725.92)	6,341.47	8,362.40	69.02 (*t*)	<0.001
	Tertiary general hospital	50,008	7,503.84 (8,858.47)	12,574.95	16,872.45		
Length of hospital stay	1–5 days	26,073	4,022.32 (3892.05)	5,563.83	5,895.97	6788.24 (*F*)	<0.001
	6–10 days	30,759	5,624.37 (4,764.85)	8,034.70	8,411.82		
	More than 10 days	21,561	11,944.24 (13,980.48)	19,324.05	22,937.42		
Surgical intervention	Had surgery	41,977	8,124.14 (10,986.93)	13,992.50	18,431.17	82.55 (*t*)	<0.001
	Did not have surgery	36,416	4,751.73 (3,916.45)	6,082.16	6,298.64		

Regarding the number of chronic diseases, inpatients with two chronic diseases had the highest medical costs (11,703.63 yuan), followed by those with three (10,425.66 yuan) and four chronic diseases (9,589.50 yuan), while inpatients with five or more chronic diseases had the lowest costs (8,804.36 yuan). The median values followed a declining trend with the number of chronic diseases, decreasing from 6,316.80 yuan for two conditions to 5,747.15 yuan for five or more conditions. The IQR also decreased progressively, from 8,514.93 yuan to 5,316.07 yuan, indicating that cost distributions became more concentrated as the number of chronic diseases increased. The differences were statistically significant (*F* = 122.78, *p* < 0.001), indicating a negative correlation between the number of chronic diseases and medical expenses.

In terms of health insurance, inpatients covered by the Urban Employee Basic Medical Insurance incurred higher average costs (11,268.02 yuan) than those covered by the Urban and Rural Resident Basic Medical Insurance (10,022.25 yuan) (*t* = 9.06, *p* < 0.001). The median values also reflected this pattern (6,625.82 yuan vs. 5,930.16 yuan), with a notably larger IQR for the Urban Employee group (7,595.53 yuan vs. 6,797.63 yuan).

Analysis of hospital level showed that inpatients in secondary general hospitals had an average cost of 6,341.47 yuan, whereas those in tertiary general hospitals had an average cost of 12,574.95 yuan (*t* = 69.02, *p* < 0.001), indicating a significant positive association between hospital level and medical costs. The median values (4,416.07 yuan vs. 7,503.84 yuan) and IQRs (3,725.92 yuan vs. 8,858.47 yuan) further highlighted the substantial differences between the two hospital levels.

For length of hospital stay, the average cost was 5,563.83 yuan for inpatients hospitalized for 1–5 days, 8,034.70 yuan for 6–10 days, and 19,324.05 yuan for more than 10 days (*F* = 6,788.24, *p* < 0.001), demonstrating that longer hospital stays were strongly associated with higher medical costs. Consistent with this, the median costs increased progressively from 4,022.32 yuan (1–5 days) to 5,624.37 yuan (6–10 days) and 11,944.24 yuan (>10 days), with a corresponding substantial expansion in IQRs, indicating greater cost variability with longer hospitalizations.

Analysis of surgical intervention revealed that inpatients without surgery incurred an average cost of 6,082.16 yuan, whereas those who underwent surgery incurred 13,992.50 yuan (*t* = 82.55, *p* < 0.001), suggesting that surgical intervention was a key determinant of medical costs. The median values (4,751.73 yuan vs. 8,124.14 yuan) and IQRs (3,916.45 yuan vs. 10,986.93 yuan) confirmed the large cost differences between the two groups.

### 3.4 Analysis of factors influencing medical expenses

The results of the multiple regression analysis indicated that total medical expenses among older inpatients were significantly influenced by multiple factors. Table 3 shows the regression analysis of factors influencing medical expenses. Among them, hospital level (*B* = 4387.29, *β* = 0.144, *t* = 43.39, *p* < 0.001) and length of hospital stay (*B* = 6641.35, *β* = 0.351, *t* = 110.84, *p* < 0.001) exerted the most pronounced effects, both showing positive associations, suggesting that inpatients treated in tertiary hospitals and those with longer hospitalizations incurred substantially higher costs. Surgical intervention was also an important factor, with surgical inpatients having significantly higher medical expenses (*B* = 6635.98, *β* = 0.225, *t* = 67.98, *p* < 0.001). Age showed a weak positive correlation with expenses (*B* = 382.16, *β* = 0.024, *t* = 7.52, *p* < 0.001), indicating a slight increase in medical costs with advancing age. The health insurance scheme had a small negative effect (*B* = −376.93, *β* = −0.011, *t* = −3.41, *p* < 0.001), suggesting that insurance somewhat reduced inpatient expenses. In particular, the number of chronic diseases was negatively associated with medical expenses (*B* = −704.67, *β* = −0.049, *t* = −15.33, *p* < 0.001), indicating that inpatients with more chronic diseases actually incurred lower costs. Variance inflation factor (VIF) values were all close to 1, suggesting that the model did not suffer from serious multicollinearity and that the regression results were relatively stable.

**Table 3:** Regression analysis of factors influencing medical expenses.

Variable	Unstandardized coefficients		Standardized coefficients	*t*	*p*	Collinearity statistics	
	*B*	Standard error	Beta (*β*)			Tolerance	VIF
Age	382.16	50.81	0.024	7.52	<0.001	0.955	1.047
Number of chronic diseases	–704.67	45.97	−0.049	−15.33	<0.001	0.976	1.025
Health insurance scheme	−376.93	110.45	−0.011	−3.41	<0.001	0.975	1.025
Hospital level	4,387.29	101.12	0.144	43.39	<0.001	0.912	1.097
Length of hospital stay	6,641.35	59.92	0.351	110.84	<0.001	0.993	1.008
Surgical intervention	6,635.98	97.62	0.225	67.98	<0.001	0.908	1.101

### 3.5 Analysis of factors influencing out-of-pocket expenses (OOPE) across various demographics

Table 4 shows that significant differences existed in OOPE across various variables. First, regarding age, OOPE decreased with increasing age (*F* = 40.14, *p* < 0.001). The average OOPE was 4673.28 yuan in the 50–59 age group, 4718.71 yuan in the 60–69 age group, 4,458.17 yuan in the 70–79 age group, and the lowest, 3,846.07 yuan, in those aged 80 years and above. The median values followed a similar declining trend, decreasing from 2,874.72 yuan (50–59 years) to 2,460.93 yuan (80 years and above). The interquartile ranges (IQRs) remained substantial across all age groups, indicating considerable variability in OOPE within each age category.

**Table 4:** Differences in out-of-pocket expenses across various variables.

Variable	Groups	Frequency	Median (Quartiles)	Mean	Standard deviation	*F/t*	*P*
Age	50–59 years	21,247	2,874.72 (3,149.07)	4,673.28	6,677.35	40.14 (F)	<0.001
	60–69 years	30,114	2,756.04 (3,210.24)	4,718.71	6,472.55		
	70–79 years	19,550	2,605.83 (2,995.98)	4,458.17	6,573.08		
	80 years and above	7,482	2,460.93 (2,458.85)	3,846.07	5,570.71		
Number of chronic diseases	2 chronic diseases	21,733	2,826.89 (3,675.90)	5,139.33	7,590.41	107.54 (F)	<0.001
	3 chronic diseases	23,874	2,733.88 (3125.93)	4,595.06	6,724.26		
	4 chronic diseases	21,568	2,684.10 (2,828.02)	4,238.97	5,678.06		
	5 or more chronic diseases	11,218	2,597.54 (2,592.99)	3,967.21	4,724.26		
Health insurance scheme	Basic Medical Insurance for Urban Employees	18,604	1,879.42 (1,559.97)	2,815.96	4,196.98	54.53 (*t*)	<0.001
	Basic Medical Insurance for Urban and Rural Residents	5,9,789	3,124.50 (3,416.56)	5,100.23	6,951.25		
Hospital level	Secondary general hospitals	28,385	1,910.69 (1,543.55)	2,624.62	3,092.15	78.85 (*t*)	<0.001
	Tertiary general hospital	50,008	3,452.06 (3,856.70)	5,655.62	7,553.19		
Length of hospital stay	1–5 days	26,073	1,928.51 (1,664.88)	2,615.60	2,835.06	5355.04 (F)	<0.001
	6–10 days	30,759	2,602.66 (2,309.35)	3,702.88	4,001.57		
	More than 10 days	21,561	5,083.07 (6,174.91)	8,127.30	10,085.56		
Surgical intervention	Had surgery	41,977	3,482.74 (4,720.16)	6,055.08	8,219.32	75.96 (t)	<0.001
	Did not have surgery	36,416	2,239.48 (1,873.87)	2,832.59	2,632.71		

The number of chronic diseases was significantly associated with OOPE (*F* = 107.54, *p* < 0.001). Inpatients with two chronic diseases had the highest average OOPE (5,139.33 yuan), whereas those with five or more chronic diseases had the lowest (3,967.21 yuan). The median values also declined progressively with the number of chronic diseases, from 2,826.89 yuan for two conditions to 2,597.54 yuan for five or more. The IQR decreased from 3,675.90 yuan to 2,592.99 yuan, suggesting that OOPE distributions became more concentrated as the number of chronic diseases increased.

Health insurance scheme also showed significant differences (*t* = 54.53, *p* < 0.001). Inpatients covered by Urban Employee Basic Medical Insurance had markedly lower OOPE (2,815.96 yuan) compared with those covered by Urban and Rural Resident Basic Medical Insurance (5,100.23 yuan). The median values (1,879.42 yuan vs. 3,124.50 yuan) and IQRs (1,559.97 yuan vs. 3,416.56 yuan) further highlighted the substantial disparity in both central tendency and dispersion between the two insurance schemes.

In terms of hospital level, inpatients in tertiary general hospitals reported much higher OOPE (5,655.62 yuan) than those in secondary general hospitals (2,624.62 yuan; *t* = 78.85, *p* < 0.001). The median values (3,452.06 yuan vs. 1,910.69 yuan) and IQRs (3,856.70 yuan vs. 1,543.55 yuan) confirmed the large differences between hospital levels.

Length of hospital stay was a decisive factor (*F* = 5355.04, *p* < 0.001). Inpatients hospitalized for 1–5 days had an average OOPE of 2,615.60 yuan, while those hospitalized for more than 10 days had the highest OOPE, averaging 8,127.30 yuan. The median values increased progressively from 1,928.51 yuan (1–5 days) to 2,602.66 yuan (6–10 days) and 5,083.07 yuan (>10 days), with IQRs expanding substantially, indicating greater OOPE variability with longer hospital stays.

Surgical intervention was also associated with significantly higher OOPE (*t* = 75.96, *p* < 0.001), with operated inpatients (6055.08 yuan) spending almost twice as much as those without surgery (2832.59 yuan). The median values (3,482.74 yuan vs. 2,239.48 yuan) and IQRs (4,720.16 yuan vs. 1,873.87 yuan) confirmed the large cost differences and greater variability among surgical patients.

### 3.6 Analysis of factors influencing out-of-pocket expenses (OOPE)

The regression analysis of factors influencing OOPE among older inpatients is presented in Table 5. The results indicated that hospital level, health insurance scheme, age, length of hospital stay, surgery, and the number of chronic diseases all had statistically significant associations with OOPE (*p* < 0.001 for all variables). Hospital level showed a positive effect, with inpatients treated in higher-level hospitals incurring an additional 2,585.36 yuan in OOPE compared with those in lower-level hospitals. Similarly, health insurance scheme was associated with increased OOPE, with inpatients covered by the Urban Employee Basic Medical Insurance paid 2,745.67 yuan less in out-of-pocket expenses than those covered by the Urban and Rural Resident Basic Medical Insurance. Age also exhibited a small but significant positive relationship, with each additional year increasing OOPE by about 100.09 yuan. The length of hospital stay was the strongest predictor, with each additional unit of stay associated with an increase of 2,620.98 yuan. Surgical intervention also significantly raised OOPE, with operated inpatients spending 2,683.01 yuan more than non-operated inpatients. Interestingly, the number of chronic diseases was negatively associated with OOPE, with each additional chronic diseases reducing OOPE by 249.91 yuan. This negative association may reflect that patients with a higher number of chronic conditions receive more conservative treatment, undergo fewer high-cost procedures, or benefit from reimbursement policies that reduce out-of-pocket expenses for certain chronic diseases. Multicollinearity diagnostics showed that tolerance values were close to 1 and VIF values were all around 1, confirming the stability and reliability of the regression model.

**Table 5:** Regression analysis of factors influencing out-of-pocket expenses.

Variable	Unstandardized coefficients		Standardized coefficients	*t*	*p*	Collinearity statistics	
	B	Standard error	Beta (β)			Tolerance	VIF
Age	100.09	22.31	0.014	4.49	<0.00	0.955	1.047
Number of chronic diseases	−249.91	20.18	−0.039	−12.38	<0.001	0.976	1.025
Health insurance scheme	2,745.67	48.49	0.180	56.63	<0.001	0.975	1.025
Hospital level	2,585.36	44.39	0.192	58.24	<0.001	0.912	1.097
Length of hospital stay	2,620.98	26.30	0.314	99.64	<0.001	0.993	1.008
Surgical intervention	2,683.01	42.86	0.207	62.61	<0.001	0.908	1.101

### 3.7 Sensitivity analysis

To assess whether the inclusion of the 50–59 age group influenced the main findings, we further restricted the analysis to inpatients aged 60 years and older (*n* = 57,146) and re-ran the multiple linear regression models. The results showed that the direction and significance of key associations remained consistent with the main analysis. For total medical expenses, the number of chronic diseases remained negatively associated with total costs (*B* = −704.67, *p* < 0.001); hospital level (*B* = 4,536.17, *p* < 0.001), length of hospital stay (*B* = 6,565.04, *p* < 0.001), and surgical intervention (*B* = 7,053.45, *p* < 0.001) remained the main positive predictors. For out of pocket expenses, the protective effect of Urban Employee Basic Medical Insurance persisted (*B* = 2,881.75, *p* < 0.001), indicating that its enrollees had significantly lower out of pocket expenses than those covered by the Urban and Rural Resident Basic Medical Insurance; the number of chronic diseases also remained negatively associated with out of pocket expenses (*B* = –249.91, *p* < 0.001). These findings suggest that the core conclusions of this study are robust to the inclusion of the 50–59 age group.

## 4 Discussion

This study was conducted in a prefecture-level city in Inner Mongolia Autonomous Region, which represents a border ethnic region in China with unique demographic and economic characteristics. Inner Mongolia, as a typical representative of China’s border ethnic regions, is characterized by vast territory, a relatively sparse population, lagging economic development, limited health resource allocation, and weaker health service capacity compared to developed coastal areas. Therefore, the findings of this study provide important insights into the financial burden of older inpatients with multimorbidity in China’s border ethnic regions, but may not be directly generalizable to coastal, urban, or more economically developed provinces. Future research in diverse regions is needed to validate and extend these findings.

### 4.1 Characteristics and implications of multimorbidity inpatients

This study revealed that inpatients aged 60–69 years represented the largest proportion of those with multimorbidity, while about one-third were aged 50–59 years. These findings indicate that multimorbidity is no longer limited to the oldest population and emphasize the need for early preventive and management strategies (36, 37). Using 30 years of follow-up data from the CARDIA cohort, Bowling et al. (2024) identified six distinct multimorbidity trajectory patterns, with some participants showing rapid accumulation of chronic conditions as early as their twenties and thirties—underscoring that the roots of multimorbidity often begin decades before traditional old age and reinforcing the importance of early risk factor modification and proactive prevention (37). Most patients presented with two to four chronic diseases, suggesting a moderate degree of multimorbidity that warrants stratified management approaches (38).

The predominance of inpatients insured under the Urban and Rural Resident Basic Medical Insurance highlights potential inequities in financial protection, as this scheme generally offers lower reimbursement rates than the Urban Employee Basic Medical Insurance. Such disparities may expose patients to higher out-of-pocket burdens and raise concerns about healthcare equity (39).

A strong preference for tertiary hospitals was also observed, reflecting both patients’ trust in high-level facilities and the limited service capacity of secondary hospitals. Strengthening county- and city-level medical institutions could improve service accessibility and help redistribute patient flow. Additionally, longer hospital stays and a high proportion of surgical cases underscore the clinical complexity of multimorbidity and the need for multidisciplinary collaboration to optimize outcomes (40).

While many studies define older adults as aged 60 or 65 years and above, this study adopted a broader definition (≥50 years) to align with the WHO SAGE framework and to capture the early phase of health transitions. This approach reflects the growing recognition that chronic disease prevention and economic burden mitigation should begin before individuals reach traditional old age. The inclusion of the 50–59 age group revealed that financial pressure associated with multimorbidity is already substantial in this younger cohort, highlighting the importance of early intervention. Although this broader definition may introduce heterogeneity in disease patterns and reimbursement rates, it also provides critical evidence for policies aimed at preventing or delaying the progression of multimorbidity into advanced age.

### 4.2 Patterns of healthcare expenses and financial burden

The results revealed that medical expenses and OOPE peaked among inpatients aged 60–69 years, but the proportion of OOPE decreased with age. The median values confirmed this pattern, with the highest median total cost also observed in the 60–69 age group (6,165.78 yuan). The substantial interquartile ranges (IQRs) across all age groups (ranging from 6,065.91 to 7,226.47 yuan for total costs) indicated considerable within-group variability. Although older inpatients incurred higher total costs, younger older-adult inpatients (50–59 years) faced a heavier relative financial burden, possibly due to lower reimbursement rates or differences in care-seeking behavior (41).

Inpatients with fewer chronic diseases incurred higher total costs, while those with more diseases had slightly lower expenses but a higher OOPE share. The median values followed a consistent declining trend, decreasing from 6,316.80 yuan for two conditions to 5,747.15 yuan for five or more conditions. Notably, the IQR also decreased progressively from 8,514.93 yuan to 5,316.07 yuan, suggesting that cost distributions became more concentrated as the number of chronic diseases increased. This may relate to treatment intensity, disease severity, or insurance coverage differences for complex multimorbidity cases (36, 38).

Significant inequities were evident between insurance schemes. The OOPE proportion was about 25% among inpatients with Urban Employee Basic Medical Insurance but over 50% among those with Urban and Rural Resident Basic Medical Insurance. Such disparities underscore the persistent inequality in healthcare financial protection (39). Treatment in tertiary hospitals and longer hospital stays substantially increased both total and OOPE, while surgical inpatients bore the highest expenses but benefited from relatively better insurance coverage.

Overall, healthcare expenses for multimorbidity inpatients were strongly influenced by age, multimorbidity level, insurance type, hospital level, length of stay, and surgical intervention. Addressing these disparities requires refined insurance design and improved efficiency of healthcare resource allocation.

### 4.3 Determinants of medical expenses

Regression analysis identified hospital level, length of stay, and surgical intervention as the main drivers of medical expenses among older inpatients. Inpatients in tertiary hospitals incurred nearly double the expenses of those in secondary hospitals, reflecting the greater clinical complexity and reliance on high-cost technologies (42). Longer hospitalizations were associated with substantially higher costs, highlighting both greater disease severity and higher consumption of medical resources (39). Similarly, surgical procedures significantly increased total expenses, consistent with the high cost of consumables, anesthesia, and postoperative care (43). A large-scale study involving 37,084 Chinese surgical patients also found that multiple disease conditions were significantly associated with higher hospitalization costs and longer hospital stays. Patients with two or more chronic diseases had higher average costs than those without comorbidities. These findings emphasize the need to enhance preoperative assessment and develop personalized care strategies to improve the treatment outcomes for patients with multiple diseases and reduce costs (44).

Although age was significantly associated with medical costs, its effect size was small. Costs peaked among inpatients aged 50–59 years and slightly declined in those aged 80 and above, possibly due to conservative treatment strategies or survival bias (45). Health insurance also exerted a moderating effect. Although inpatients with Basic Medical Insurance for Urban Employees had higher total hospitalization costs than those with Basic Medical Insurance for Urban and Rural Residents, their out-of-pocket expenses were lower after insurance reimbursement, suggesting that insurance coverage reduces cost escalation and mitigates patient burden (46). This finding aligns with a recent review of China’s social health insurance system, which noted that while China has achieved universal health coverage and reduced out-of-pocket expenditures over the past three decades, the rapidly aging population is placing considerable pressure on the financial sustainability of these schemes (47). Maintaining a balance between expanding healthcare benefits and ensuring financial sustainability will be essential for the continued success of the system (47).

A negative association was observed between the number of chronic diseases and total costs, contrasting with the conventional view that more conditions lead to higher expenses (48). This pattern may be explained by several factors. First, patients with a higher number of chronic conditions may receive more conservative management approaches, particularly in frail older individuals, resulting in fewer high-cost procedures and shorter therapeutic interventions. Second, the use of only the first five diagnoses may truncate the actual disease burden, potentially attenuating the cost estimates for patients with complex multimorbidity. Third, patients with multiple chronic conditions may be more likely to be admitted under a primary diagnosis that is relatively low-cost (e.g., hypertension), while those with fewer conditions may have a primary diagnosis that requires costly interventions (e.g., surgery). Future research should further explore this atypical relationship by employing disease-cluster analyses and incorporating measures of disease severity, functional status, and treatment intensity to better understand the economic implications of multimorbidity and inform more targeted policy interventions (49).

### 4.4 Determinants of out-of-pocket expenses (OOPE)

OOPE among older inpatients was primarily influenced by hospital level, length of stay, surgical intervention, and insurance type. Inpatients in tertiary hospitals bore significantly higher OOPE, reflecting the greater intensity and technological sophistication of services provided (42). The median OOPE for tertiary hospitals (3,452.06 yuan) was nearly double that of secondary hospitals (1,910.69 yuan), with a substantially larger IQR (3,856.70 yuan vs. 1,543.55 yuan), indicating both higher costs and greater variability in tertiary settings. Prolonged hospitalization strongly increased OOPE, emphasizing the importance of reducing unnecessary inpatient days (39). The median OOPE increased progressively from 1,928.51 yuan (1–5 days) to 5,083.07 yuan (>10 days), with a corresponding expansion in IQR from 1,664.88 yuan to 6,174.91 yuan, highlighting the growing variability associated with longer stays. Surgical treatment also substantially raised OOPE, given the costs of anesthesia, consumables, and postoperative monitoring (49). The median OOPE for surgical patients (3,482.74 yuan) was more than 1.5 times that of non-surgical patients (2,239.48 yuan), and the IQR for surgical patients (4,720.16 yuan) was more than double that of non-surgical patients (1,873.87 yuan), reflecting the greater cost dispersion among those undergoing surgery.

Insurance coverage exhibited a clear gradient. The Urban Employee Basic Medical Insurance enrollees experienced markedly lower OOPE than those with Urban Employee Basic Medical Insurance, highlighting persistent inequities in financial protection (50). The median OOPE for Urban Employee enrollees (1,879.42 yuan) was only 60% of that for Urban–Rural Resident enrollees (3,124.50 yuan), and the IQR for Urban Employee enrollees (1,559.97 yuan) was less than half that of Urban–Rural Resident enrollees (3,416.56 yuan), indicating that the former group not only had lower out-of-pocket costs but also more predictable expenses. OOPE tended to decline with age, possibly reflecting more conservative clinical management and higher reimbursement for older patients (51). The negative association between the number of chronic diseases and OOPE may indicate both insurance protection for chronic conditions and the shift toward less invasive care for multimorbid patients (48).

These findings underscore that OOPE is shaped by both healthcare utilization patterns and systemic features of the insurance and hospital systems. Strengthening the financial protection function of basic medical insurance, particularly for inpatients covered under resident schemes, and promoting efficient hospital resource use are essential to reducing the financial burden of multimorbidity among the older individuals.

### 4.5 Recommendations

Several policy measures are recommended to alleviate the financial burden of multimorbidity among older inpatients and to promote greater equity and efficiency within China’s healthcare system. First, systematic early prevention and health promotion initiatives should target middle-aged and younger older adults. Second, the tiered healthcare delivery system should be strengthened through expansion of the service capacity and clinical capabilities of secondary hospitals. Third, health insurance benefit structures should be optimized to reduce disparities across insurance schemes. Fourth, hospitalization expenses should be controlled via evidence-based management of length of stay and surgical indications. Finally, the implementation of integrated, multidisciplinary care models for patients with multimorbidity should be prioritized, alongside the expansion of long-term care and rehabilitation services.

### 4.6 Limitation

This study has several limitations. First, this study used retrospective inpatient data from six hospitals in a single city in Inner Mongolia Autonomous Region. Although the random sampling procedure minimized selection bias within the city, the findings may not be directly generalizable to other provinces, rural facilities, or more developed regions. Nevertheless, Inner Mongolia is a typical representative of China’s border ethnic regions, and the results of this study provide important insights for understanding the financial burden of older inpatients with multimorbidity in such areas. This study should be viewed as a city-level case study that offers insights for China’s border ethnic regions. Second, only inpatient costs and out-of-pocket expenses were analyzed, excluding outpatient, indirect, and long-term care costs that are important components of the total economic burden for inpatients with multimorbidity. Although the proportion of out-of-pocket expenses in descriptive analyses was reported, the OOPE share was not formally modeled as an outcome, nor were catastrophic expenditure, household income, or impoverishment risk assessed. Future research could employ fractional regression or logit-transformed models to examine factors influencing the share of costs borne by patients, as this metric more directly reflects financial protection, and incorporate measures of household economic impact to better capture the full financial burden of multimorbidity. Third, multimorbidity was measured solely by the count of chronic diseases, without considering disease severity, specific disease combinations, or functional status. This simplified measure may not capture the substantial cost variations associated with different disease clusters or the intensity of clinical management. The absence of such granular data limits the clinical and policy applicability of the findings, as interventions and resource allocation often need to target specific disease combinations or patient functional profiles. Fourth, the cross-sectional design restricts causal inference between inpatient characteristics, multimorbidity, and medical expenses. Future longitudinal research should explore how multimorbidity evolves over time and how policy interventions affect healthcare costs and financial protection. Fifth, the inclusion of patients aged 50–59 in the study sample may introduce heterogeneity, as this age group may differ from older age groups in terms of disease patterns, healthcare utilization, and reimbursement rates. This heterogeneity should be considered when interpreting the findings. Future studies could benefit from age-stratified analyses to better understand age-specific differences in financial burden. Sixth, the regression models did not adjust for several potentially important confounders, including diagnosis category, disease severity, emergency versus elective admission, ICU use, and discharge status. These factors may influence both treatment intensity and cost outcomes. Additionally, patients were nested within only six hospitals, and hospital-specific pricing or practice patterns may introduce clustering effects that were not accounted for in the current analysis. Future research with richer clinical data and multi-level modeling approaches could address these limitations. Seventh, the cost data exhibited right-skewness, with standard deviations larger than means. Although *t*-tests, ANOVA, and ordinary linear regression are commonly used in health economics research, they may not be optimal for highly skewed cost data. Future studies should consider using generalized linear models with a gamma distribution and log link, or quantile regression approaches, to provide more robust estimates.

## 5 Conclusion

This study highlights the high prevalence and substantial economic impact of multimorbidity among older inpatients in Inner Mongolia, China. Multimorbidity is most common among individuals aged 60–69 years, with a notable trend toward earlier onset. Total medical costs and out-of-pocket expenses are primarily driven by hospital level, length of stay, and surgical intervention, while insurance type, age, and chronic disease combinations also play significant roles. Importantly, inpatients enrolled in the Urban and Rural Resident Basic Medical Insurance bear a considerably heavier financial burden than those covered by the Urban Employee Basic Medical Insurance, revealing persistent inequities in financial protection.

These findings underscore the need for comprehensive strategies to address the challenges posed by multimorbidity. Strengthening early prevention, improving tiered healthcare delivery, optimizing insurance benefit design, enhancing cost control, and promoting multidisciplinary care are essential to improving equity and sustainability in healthcare financing of older inpatients. This study reveals the high prevalence and substantial economic burden of multimorbidity among older inpatients in a city in Inner Mongolia Autonomous Region, providing an important case reference for understanding the financial burden of multimorbidity in China’s border ethnic regions.

## Data Availability

The data analyzed in this study is subject to the following licenses/restrictions: the datasets analyzed in this study were provided by the local healthcare insurance bureau under a data-sharing agreement that restricts public dissemination to protect patient privacy and comply with institutional regulations. Therefore, the raw data are not publicly available. However, anonymized or aggregated data may be made available from the corresponding author upon reasonable request, subject to approval by the data provider and the ethics committee of the participating institutions. Requests to access these datasets should be directed to BX, xianbensong@163.com.
