# Supplementary material for: Financial burden of older inpatients with multimorbidity in China: a cross-sectional study
Source: Front Public Health. 2026 Apr 30;14:1767499. doi: 10.3389/fpubh.2026.1767499 (PMC13171543; doi:10.3389/fpubh.2026.1767499)
Supplement: Supplementary file 1 [file Table_1.docx]

**Supplementary Table S1.** List of 46 chronic conditions and corresponding ICD-10 codes.

| **No.** | **Chronic conditions** | **ICD-10 codes** |
| --- | --- | --- |
| 1 | Hypertension | I10–I15 |
| 2 | Lipid metabolism disorders | E78 |
| 3 | Chronic low back pain | M40–M45, M47, M48.0–M48.2, M48.5–M48.9 M50–M54 |
| 4 | Severe vision reduction | H17–H18, H25–H28, H31, H33,H34.1–H34.2, H34.8–H34.9,H35–H36, H40, H43, H47, H54 |
| 5 | Joint arthrosis | M15–M19 |
| 6 | Diabetes mellitus | E10–E14 |
| 7 | Chronic ischemic heart disease | I20, I25, I21 |
| 8 | Thyroid diseases | E01–E05, E06.1–E06.3, E06.5, E06.9, E07 |
| 9 | Cardiac arrhythmias | I44–I45, I46.0, I46.9, I47–I48,  I49.1–I49.9 |
| 10 | Obesity | E66 |
| 11 | Hyperuricemia/gout | E79, M10 |
| 12 | Prostatic hyperplasia | N40 |
| 13 | Lower limb varicosis | I83, I87.2 |
| 14 | Liver disease | K70, K71.3–K71.5, K71.7, K72.1, K72.7, K72.9, K73–K74, K76 |
| 15 | Depression | F32–F33 |
| 16 | Asthma/COPD | J40–J45, J47 |
| 17 | Gynecological problems | N81, N84–N90, N93, N95 |
| 18 | Atherosclerosis/PAOD | I65–I66, I67.2, I70, I73.9 |
| 19 | Osteoporosis | M80–M82 |
| 20 | Renal insufficiency | N18–N19 |
| 21 | Cerebral ischemia/chronic stroke | I60–I64, I69, G45 |
| 22 | Cardiac insufficiency | I50 |
| 23 | Severe hearing loss | H90, H91.0, H91.1, H91.3, H91.8, H91.9 |
| 24 | Chronic cholecystitis/gallstones | K80, K81.1 |
| 25 | Somatoform disorders | F45 |
| 26 | Hemorrhoids | K64 |
| 27 | Intestinal diverticulosis | K57 |
| 28 | Rheumatoid arthritis/chronic  polyarthritis | M05–M06, M79.0 |
| 29 | Cardiac valve disorders | I34–I37 |
| 30 | Neuropathies | G50–G64 |
| 31 | Dizziness | H81–H82, R42 |
| 32 | Dementia | F00–F03, F05.1, G30, G31, R54 |
| 33 | Urinary incontinence | N39.3–N39.4, R32 |
| 34 | Urinary tract calculi | N20 |
| 35 | Anemia | D50–D53, D55–D58, D59.0–D59.2, D59.4–D59.9, D60.0, D60.8, D60.9, D61, D63–D64 |
| 36 | Anxiety | F40–F41 |
| 37 | Psoriasis | L40 |
| 38 | Migraine/chronic headache | G43, G44 |
| 39 | Parkinson’sdisease | G20–G22 |
| 40 | Cancer | C00–C14, C15–C26, C30–C39,  C40–C41, C43–C44, C45–C49,  C50, C51–C58, C60–C63,  C64–C68, C69–C72, C73–C75,  C76–C80,C81–C96, C97,  D00–D09, D37–D48, |
| 41 | Allergy | H01.1, J30, L23, L27.2, L56.4,K52.2, K90.0, T78.1, T78.4, T88.7 |
| 42 | Chronic gastritis/GERD | K21, K25.4–K25.9, K26.4–K26.9,  K27.4–K27.9, K28.4–K28.9,  K29.2–K29.9 |
| 43 | Sexual dysfunction | F52, N48.4 |
| 44 | Insomnia | G47, F51 |
| 45 | Tobacco abuse | F17 |
| 46 | Hypotension | I95 |
